# The effect of acidity on the physicochemical properties of two hydraulic calcium silicate-based cements and two calcium phosphate silicate-based cements

**DOI:** 10.1186/s12903-023-03211-8

**Published:** 2023-08-11

**Authors:** Yan Yang, He Liu, Zhe-Jun Wang, Pei Hu, Markus Haapasalo, Adriana Manso, Jing-Zhi Ma, Ya Shen

**Affiliations:** 1 Department of Stomatology, Tongji Hospital, Tongji Medical College, Huazhong University of Science and Technology, Wuhan, 430030 Hubei Province China; 2 Division of Endodontics, Department of Oral Biological and Medical Sciences, Faculty of Dentistry, University of British Columbia, 2199 Wesbrook Mall, Vancouver, BC V6T 1Z3 Canada; 3 Division of Restorative Dentistry, Department of Oral Health Sciences, Faculty of Dentistry, University of British Columbia, Vancouver, Canada

**Keywords:** Butyric acid, Calcium phosphate silicate-based cements, Calcium silicate-based cements, Phosphate-buffered saline, Physicochemical properties

## Abstract

**Background:**

Bioceramic cements have been widely used in endodontic treatment. This study aimed to compare the microhardness, elastic modulus, internal microstructure and chemical compositions of Biodentine, WMTA, ERRM Putty, iRoot FS and IRM after exposure to PBS, butyric acid, and butyric acid followed by PBS.

**Methods:**

Specimens of each material were prepared and randomly divided into 5 subgroups (n = 5): subgroup A: PBS (pH = 7.4) for 4 days, subgroup B: PBS (pH = 7.4) for 14 days, subgroup C: butyric acid (pH = 5.4) for 4 days, subgroup D: butyric acid (pH = 5.4) for 14 days, subgroup E: butyric acid for 4 days followed by 10 days in contact with PBS. The surface microhardness, elastic modulus, internal morphologic and chemical compositions of specimens were analyzed.

**Results:**

The microhardness and elastic modulus values of all materials were significantly higher in the presence of PBS compared to exposure to butyric acid, with the same setting time (*P* < 0.01). After 4-day exposure to butyric acid followed by 10-day exposure to PBS, the microhardness values returned to the same level as 4-day exposure to PBS (*P* > 0.05). Biodentine showed significantly higher microhardness and elastic modulus values than other materials, while IRM displayed the lowest (*P* < 0.01).

**Conclusion:**

Biodentine seems the most suitable bioceramic cements when applied to an infected area with acidic pH. Further storage at neutral pH, e.g. PBS reverses the adverse effects on bioceramic cements caused by a low pH environment.

## Background

Bioceramic cements have been widely used as both root repair materials and dentin substitutes to seal the communication between the root canal and periodontal environments or the oral cavity in several clinical situations, such as root canal filling, retrofilling, perforation repair, apical barrier formation, direct pulp capping, pulpotomy, and regenerative endodontic procedures [1–7]. However, when the surrounding tissues at the target site are involved in inflammation, cements are usually placed in an acidic environment, where the normal physiological pH of 7.4 decreases to an acidic level [8]. Low pH has been shown to affect the physical and chemical properties of some cements [9–17].

Hydraulic calcium silicate-based cements (HCSCs) are the most popular bioceramic cements for endodontic applications. The first patented and typical representative of this group is mineral trioxide aggregate (MTA; Dentsply Tulsa Dental Specialties, Johnson City, TN, USA). Despite its favorable properties and gold standard material status, MTA has been reported to have reduced hardness, increased solubility, decreased push-out bond strength to dentine, impaired sealing ability, and weakened ultrastructure in low pH situations [9–17]. To overcome the limitations of MTA, Biodentine (Septodont, Saint-Maur-des-fossés Cedex, France) was marketed in 2009. Biodentine has improved handling characteristics and a shorter setting time than MTA, and it has the added advantage of being less likely to cause tooth discoloration. However, when exposed to an acidic environment, Biodentine has been found to have impaired push-out bond strength [16]. Nonetheless, Biodentine has been shown to be less sensitive and to have higher surface hardness, compressive strength, and bond strength than white MTA (WMTA; Dentsply Tulsa Dental Specialties), despite a change in the microstructure of both cements [11]. Interestingly, it has been reported that MTA shows better sealing ability than Biodentine when tested using a fluid transport model after exposure to citric acid [17].

Calcium phosphate silicate-based cements (CPSCs) are novel endodontic repair bioceramic materials composed of hydraulic calcium silicates and phosphate salts [2]. These cements have similar clinical uses as MTA, and the intention behind their development was to improve their biocompatibility, bioactivity, and mechanical properties by reacting the phosphates and calcium hydroxides (CH) produced during calcium silicates hydration [18]. Two commercially available CPSCs are EndoSequence Root Repair Material Putty (ERRM Putty; Brasseler USA, Savannah, GA, USA) and iRoot Fast Set Root Repair Material (iRoot FS; Innovative Bioceramix, Vancouver, Canada). These premixed, ready-to-use, non-shrinking, insoluble, radiopaque, aluminum-free, white hydraulic cements only set in an aqueous environment and have similar biocompatibility to MTA, but with better handling properties [2, 19, 20]. The iRoot FS has a faster setting time and hydrating process than ERRM Putty and white MTA, and comparable mechanical properties to WMTA [2]. Previous studies have shown that when ERRM Putty and WMTA were exposed to an acidic environment, they exhibited reduced microhardness, more porous and less crystalline microstructures, and reduced push-out bond strength [12, 21, 22]. However, there is currently no information available on the effect of acidic pH on the microhardness and microstructure of iRoot FS, the novel CPSC.

When bioceramic cements are placed in contact with existing infection and inflammation, they may be exposed to an acidic pH. However, after the inflammatory process is controlled through therapeutic interventions and the inflamed tissue is eliminated, the tissue pH returns to normal within seven days or less [9, 10]. The purpose of this study was to evaluate the changes in microhardness, microstructure, and phase compositions using X-ray diffraction of two HCSCs (WMTA and Biodentine) and two CPSCs (ERRM Putty and iRoot FS) when exposed to different environment including phosphate-buffered saline (PBS), an acidic media butyric acid, and exposure to butyric acid followed by PBS. The last scenario was to determine if storing the materials in PBS could reverse any changes induced by low pH. Intermediate restorative material (IRM; Dentsply Caulk, Milford, DE, USA) was used as a reference material.

## Methods

### Specimen preparation

The materials used in this study included two commercial CPSCs, Biodentine and WMTA, as well as two HCSCs, ERRM Putty and iRoot FS. IRM was used as a control material. Table 1 shows compositions of the materials in this study [23–26]. All materials were prepared and manipulated according to the manufacturers’ instructions. Each material group containing 25 specimens was divided into five subgroups (n = 5) based on the storage media and duration of exposure. The subgroups included exposure to calcium-free PBS (Gibco, Thermo Fisher, Waltham, MA, USA) at a pH of 7.4 for 4 days (subgroup A) or 14 days (subgroup B), exposure to butyric acid (Sigma-Aldrich, St Louis, MO, USA) at a pH of 5.4 for 4 days (subgroup C) or 14 days (subgroup D), or exposure to 1 mmol/L butyric acid for 4 days followed by 10 days in contact with PBS (subgroup E). The materials were placed into cylindrical rubber molds with a diameter of 5 mm and a thickness of 2 mm using minimal pressure [27]. The molds were then wrapped in gauze soaked with PBS or butyric acid and placed in a 37 °C incubator in a 100% relative humidity environment. The fresh material before setting was packed into the molds and kept in the mold until the exposure duration was done. The pH level of the gauze was maintained by replacing it every 12 h [10].

**Table 1:** Compositions of two HCSCs (Biodentine and WMTA), two CPSCs (ERRM Putty and iRoot FS) and IRM used in this study

Products	Manufacturer	City country	Compositions
Biodentine	Septodont	Saint-Maur-des-fossés Cedex, France	Powder: tricalcium silicate, dicalcium silicate, calcium carbonate, iron oxide, and zirconium oxide. Liquid: water with calcium chloride and soluble polymer (polycarboxylate) [23]
WMTA	Dentsply Tulsa Dental Specialties	Johnson City, TN, USA	Powder: tricalcium aluminate, bismuth oxide, tricalcium silicate, dicalcium silicate, gypsum Liquid: distilled water [24]
ERRM Putty	Brasseler USA	Savannah, GA, USA	Tricalcium silicate, dicalcium silicate, calcium phosphate monobasic, calcium hydroxide, colloidal silica, water-free thickening agent [23]
iRoot FS	Innovative Bioceramix	Vancouver, BC, Canada	Calcium silicates, zirconium oxide, tantalum pentoxide, calcium phosphate monobasic, anhydrous calcium sulphate and filler agents [25]
IRM	Dentsply Caulk	Milford, DE, USA	Zinc Oxide, eugenol, polymethylmethacrylate [26]

Abbreviations: CPSCs: calcium phosphate silicate-based cements; HCSCs: hydraulic calcium silicate-based cements; ERRM Putty: EndoSequence Root Repair Material Putty; IRM: intermediate restorative material; iRoot FS: iRoot Fast Set Root Repair Material; WMTA: white mineral trioxide aggregate

### Microhardness and elastic modulus analyses

The dynamic microhardness (DH) and elastic modulus (E) of the materials were evaluated using a dynamic ultra-micro-hardness tester (DUH-211 S; Shimadzu Co., Kyoto, Japan) equipped with a Berkovich indenter (115° triangular pyramid-shaped). Three samples were prepared for each subgroup. Once the experimental setup was completed, all specimens were demolded and their surfaces were wet-polished with minimal hand pressure, using 800, 1,200, 2,400, and 4,000 grit silicon carbide sandpapers for 30 s each.

During the load-unload test, the load force was continuously increased and decreased at a constant speed ranging from 0 to 100 mN. The indenter was held at the maximum load and minimum load for 10 s and 5 s, respectively. The test force (F) and indentation depth (h) were automatically recorded as the indenter pressed against the specimen. The recorded data were then used to generate a force-depth curve, from which the dynamic microhardness (calculated using Eq. 1) and elastic modulus (calculated using Eq. 2) were obtained and collected by the software. Both equations were obtained from the manufacturer’s manual for the dynamic ultra-micro-hardness tester.

1) DH = α × F/h^2^

where *α* is a geometrical constant of the Berkovich indenter (3.8584), *F* is the load during the test and *h* is the penetration depth of indentation. The unit for this hardness expression is kgf/mm^2^ which is normally not used.

2) 1/Er = (1-V^2^)/E + (1-Vi^2^)/Ei

where *Er* is the reduced modulus of the indentation contact, *V* is Poisson’s ratio of the sample, *Vi* is Poisson’s ratio of the Berkovich indenter (0.07), and *Ei* is the modulus of the indenter (1.14 *×* 10^6^ N/mm^2^).

In this study, indentations were randomly made on the polished surface, with 0.5 mm between each indentation and the specimen periphery, until ten representative test force and depth curves were obtained for each specimen [28]. A total of thirty indentation results were collected for each subgroup, and the mean DH and E values for each subgroup were calculated based on the collected data.

### Scanning electron microscope and X-ray energy dispersive analyses

To analyze the internal microstructure of the set samples in each experimental subgroup, scanning electron microscope (SEM) (Helios Nanolab 650; FEI, Eindhoven, Netherlands) analysis was employed, and X-ray energy dispersive spectroscope (EDS) analysis was carried out for element analysis. The specimens, which were prepared using the same protocol for each experimental subgroup, were sectioned into two halves using a disposable surgical scalpel blade. The samples were then dehydrated using increasing concentrations of ethanol and were subsequently dried using a critical point drier (Samdri-795; Tousimis Research Corporation, Rockville, MD, USA). Next, the samples were attached to aluminum stubs, and the longitudinally fractured surfaces were coated with iridium and examined using SEM. Additionally, EDS was used to analyze both the general area and specific shaped crystals.

### X-ray diffraction analysis

The set materials, which were exposed to various environmental conditions, were crushed into a fine powder using an agate mortar and pestle after being dried in a vacuum desiccator. X-ray diffraction (XRD) was then performed to analyze the powder’s phases. The diffractometer used for the XRD analysis was a Rigaku multiflex diffractometer (Rigaku Japan Corporation, Tokyo, Japan) that used Cu Kα radiation at 40 kV and 20 mA. The detector was rotated between 15 and 45°, with a sampling width of 0.01° and a scan speed of 1°/min. Phase identification was conducted using Match! software (Crystal Impact GbR, Kreuzherrenstr., Bonn, Germany) and the Crystallography Open Database (COD), which is free of charge [29].

### Statistical analysis

The data analysis was performed using SPSS 22.0 software (IBM SPSS Inc., Chicago, IL, USA). Levene’s test was performed to assess the homogeneity of variance, and Kolmogorov–Smirnov test was used to calculate the normality of distribution. Since the variance was uneven or the data did not conform to a normal distribution, the nonparametric test Kruskal-Wallis analysis of variance followed by Mann-Whitney U test was used to identify the statistically significant difference between the average DH (or E) values of each subgroup which was the same material, as well as the average DH (or E) values of each material at the significance level of α = 0.05.

## Results

### Microhardness and elastic modulus findings

**Fig. 1:**
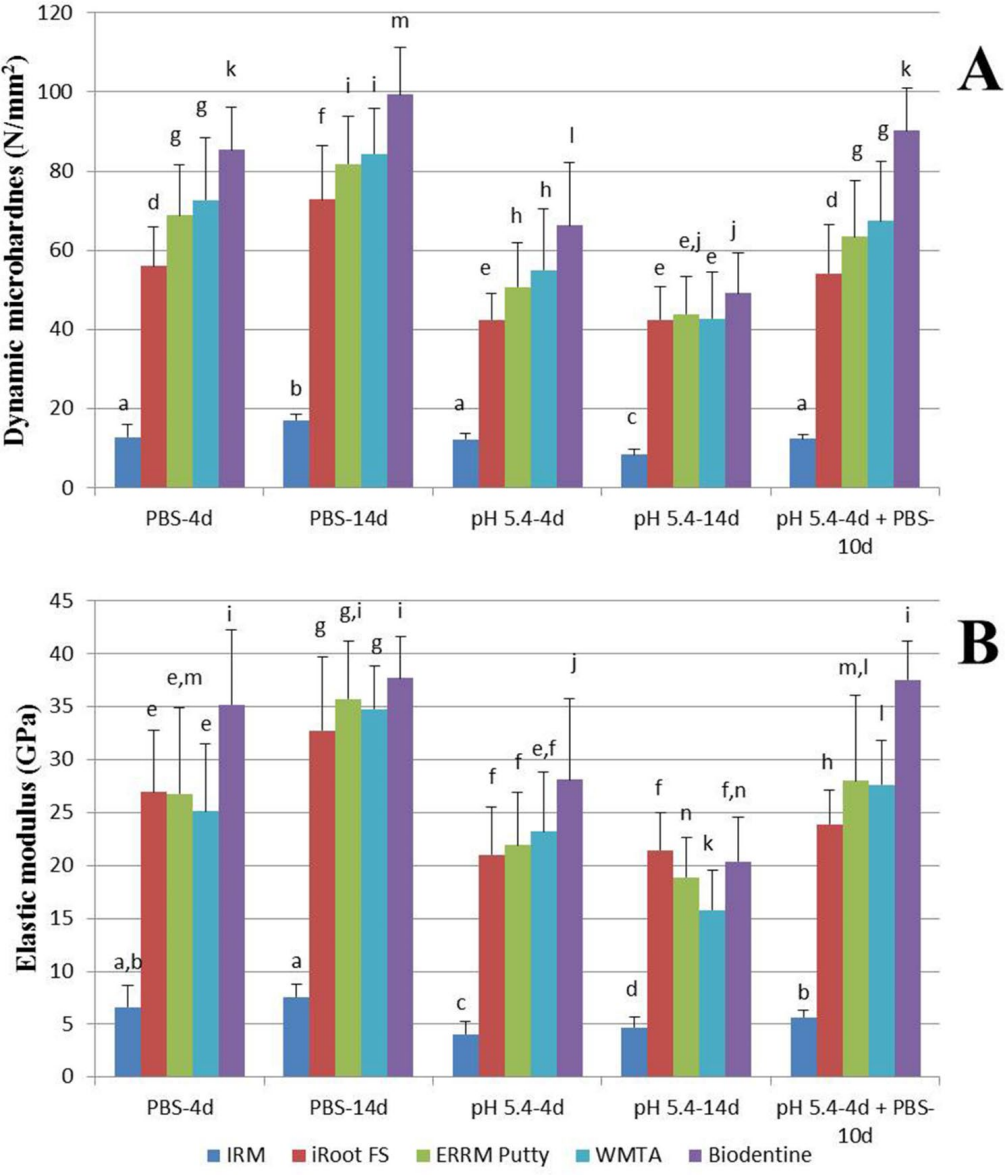
Mean surface dynamic microhardness and elastic modulus of different root repair materials after setting in various environmental conditions. Distinct superscript lower-case letters above each bar graph signify a statistically significant dissimilarity among the subgroups represented by the respective bar graph (*P* < 0.01), while those with equal letters mean no significant difference (*P* > 0.05). Abbreviations: ERRM Putty: EndoSequence Root Repair Material Putty; IRM: intermediate restorative material; iRoot FS: iRoot Fast Set Root Repair Material; WMTA: White mineral trioxide aggregate

Figure 1 presents the results of microhardness (A) and elastic modulus (B) tests. The dynamic microhardness and elastic modulus values were significantly higher at neutral pH in PBS than when exposed to butyric acid (pH 5.4) for all bioceramic materials at the same setting time (*P* < 0.01). After 10-day exposure to PBS following 4-day exposure to butyric acid, the microhardness values returned to the same level as 4-day exposure to PBS (*P* > 0.05), and an increase in elastic modulus values was observed. In PBS, the microhardness and elastic modulus of all materials were significantly higher for 14-day exposure than for 4-day exposure (*P* < 0.01), whereas 14-day exposure to butyric acid resulted in lower microhardness and elastic modulus than 4-day exposure (*P* < 0.01), except for iRoot FS (*P* > 0.05). Under all experimental conditions, IRM had the lowest microhardness (12.86 ± 3.01, 17.10 ± 1.33, 12.28 ± 1.47, 8.32 ± 1.35, 12.34 ± 1.13 for each subgroup) and elastic modulus (6.62 ± 2.07, 7.54 ± 1.22, 4.08 ± 1.15, 4.71 ± 1.02, 5.68 ± 0.62 GPa for each subgroup), while Biodentine had the highest (microhardness 85.39 ± 10.86, 99.33 ± 11.95, 66.24 ± 15.93, 49.17 ± 10.21, 90.23 ± 10.72 and elastic modulus35.20 ± 7.04, 37.74 ± 3.92, 28.12 ± 7.68, 20.38 ± 4.18, 37.58 ± 3.64 GPa for each subgroup) (*P* < 0.01). No significant differences in microhardness were found between ERRM Putty and WMTA (*P* > 0.05), but both had higher values than iRoot FS (*P* < 0.01). The elastic modulus values of iRoot FS, ERRM Putty, and WMTA did not show a statistically significant difference (*P* > 0.05). All HCSCs and CPSCs showed increased microhardness as time progressed when stored in PBS. However, in an acidic environment, the microhardness value of these cements decreased over time, except for iRoot FS. A similar trend was observed for elastic modulus.

### SEM and EDS findings

**Fig. 2:**
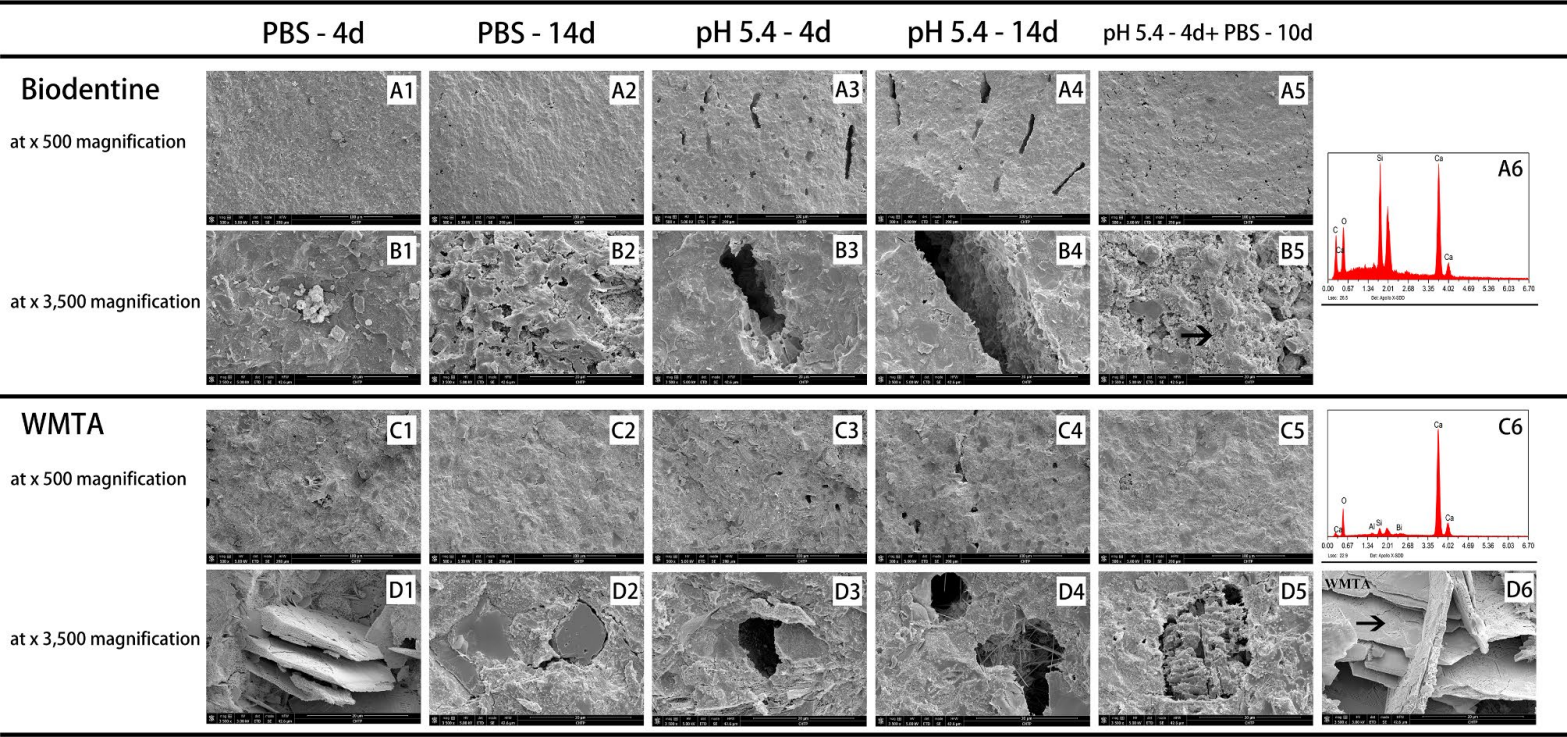
SEM images of cross-sections of two HCSCs: Biodentine (A1 - A5, B1 - B5) and WMTA (C1 - C5, D1 - D6), exposed to various environmental conditions: (A1 - D1) after 4 days setting with PBS (pH 7.4); (A2 - D2) after 14 days setting with PBS (pH 7.4); (A3 - D3) after 4 days setting with butyric acid (pH 5.4); (A4 - D4) after 14 days setting with butyric acid (pH 5.4); and (A5 - D5) after 4 days setting with butyric acid (pH 5.4) followed by 10 days setting with PBS. (A1 - A5, C1 - C5, × 500 magnification). (B1 - B5, D1 - D6, × 3,500 magnification). SEM images (B5, D6, ×3,500 magnification) of crystals in cross-section of each bioceramic cement and corresponding EDS analysis plots (A6, C6). Abbreviations: HCSCs: hydraulic calcium silicate-based cements; PBS: phosphate-buffered saline; SEM: scanning electron microscope; WMTA: white mineral trioxide aggregate

**Fig. 3:**
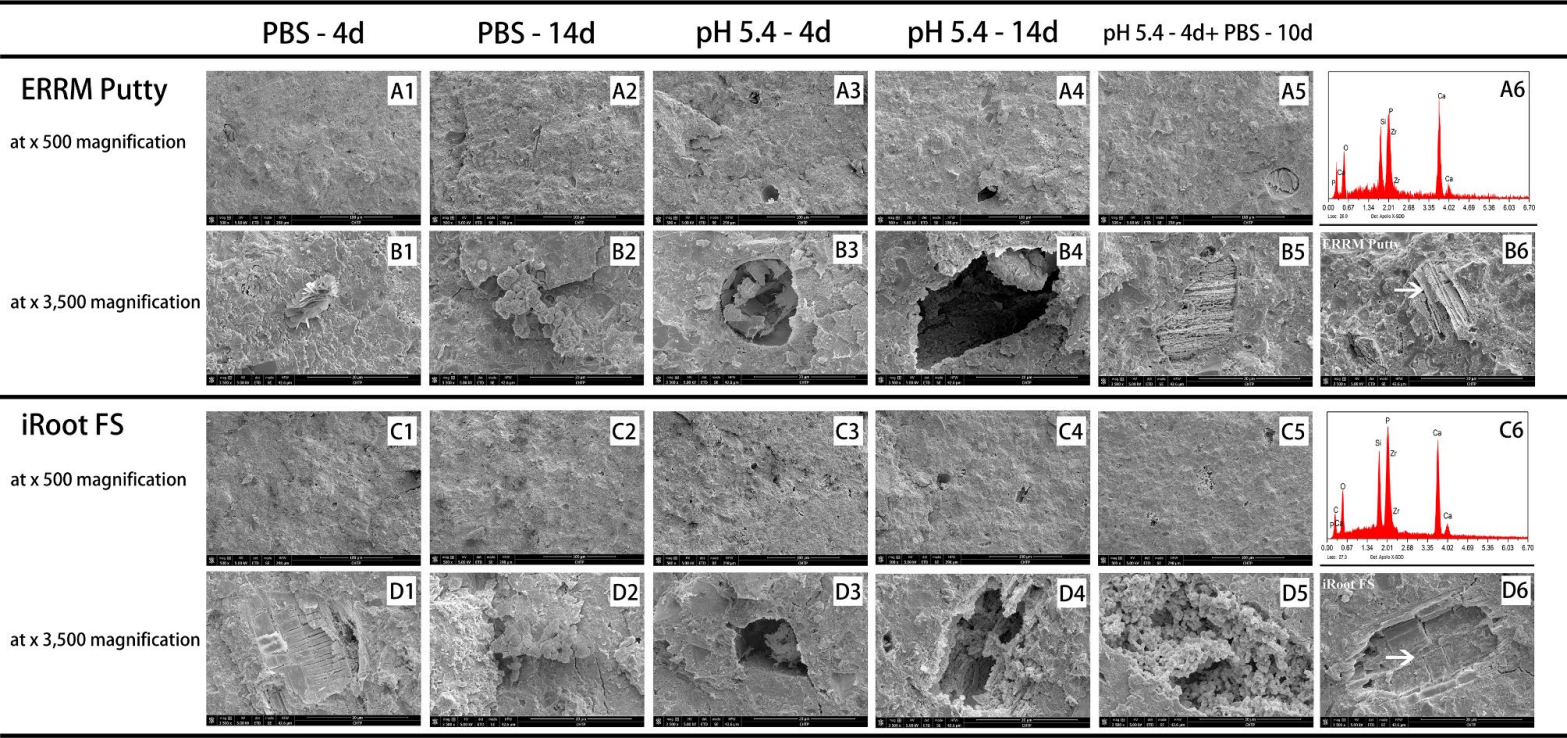
SEM images of cross-sections of two CPSCs: ERRM Putty (A1 - A5, B1 - B6) and iRoot FS (C1 - C5, D1 - D6), exposed to various environmental conditions: (A1 - D1) after 4 days setting with PBS (pH 7.4); (A2 - D2) after 14 days setting with PBS (pH 7.4); (A3 - D3) after 4 days setting with butyric acid (pH 5.4); (A4 - D4) after 14 days setting with butyric acid (pH 5.4); and (A5 - D5) after 4 days setting with butyric acid (pH 5.4) followed by 10 days setting with PBS. (A1 - A5, C1 - C5, × 500 magnification). (B1 - B6, D1 - D6, × 3,500 magnification). SEM images (B6, D6, ×3,500 magnification) of crystals in cross-section of each bioceramic cement and corresponding EDS analysis plots (A6, C6). Abbreviations: CPSCs: calcium phosphate silicate-based cements; ERRM Putty: EndoSequence Root Repair Material Putty; iRoot FS: iRoot Fast Set Root Repair Material; PBS: phosphate-buffered saline; SEM: scanning electron microscope

**Fig. 4:**
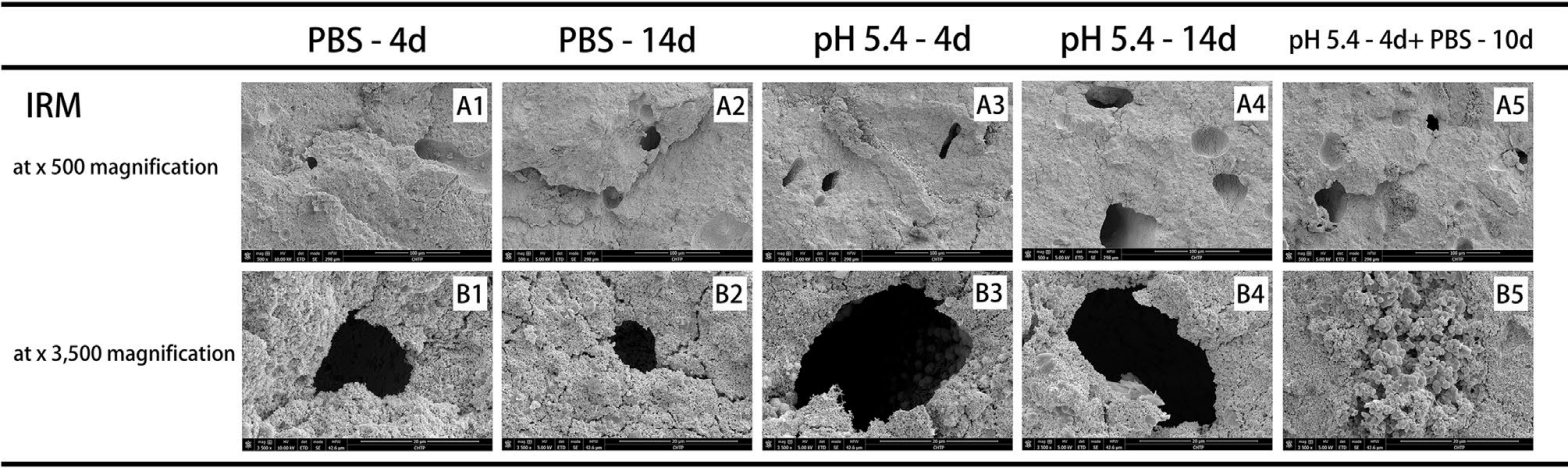
SEM images of cross-sections of IRM exposed to various environmental conditions: (A1 - B1) after 4 days setting with PBS (pH 7.4); (A2 - B2) after 14 days setting with PBS (pH 7.4); (A3 - B3) after 4 days setting with butyric acid (pH 5.4); (A4 - B4) after 14 days setting with butyric acid (pH 5.4); and (A5 - B5) after 4 days setting with butyric acid (pH 5.4) followed by 10 days setting with PBS. (A1 - A5, × 500 magnification). (B1 - B5, × 3,500 magnification). Abbreviations: IRM: intermediate restorative material; PBS: phosphate-buffered saline; SEM: scanning electron microscope

The internal microstructure of all test materials was examined using SEM after exposure to various storage media for different time periods (Fig. 2A1-5, B1-5, C1-5, D1-6, Fig. 3A1-5, B1-6, C1-5, D1-6, and Fig. 4A1-5, B1-5). In general, specimens exposed to butyric acid (pH 5.4) exhibited more porous structures and larger pores than those exposed to PBS under the same magnification. Additionally, microchannels were observed (Fig. 2A3, A4, and Fig. 4A3). Samples kept in PBS showed more crystallized structures than those in the acid condition. After exposure to PBS for 10 days following 4 days of butyric acid exposure, the number and size of voids decreased, and crystal-like structures filled them again.

Biodentine samples stored in PBS showed the appearance of spherical precipitates composed of small, fusiform-shaped crystals, and globular aggregate particles were embedded in the material (Fig. 2B2). These structures were less likely to be found in Biodentine exposed to butyric acid. However, when Biodentine was restored in PBS after exposure to acid, the pores were filled with aggregates of globular particles (Fig. 2B5). Photomicrographs of WMTA specimens revealed laminated cross-stratified structures and bundles of jagged needle-like formations (Fig. 2D1 - D5). In contrast, when WMTA was exposed to butyric acid, only partial or no laminar plate-like structures without well-defined edges were detected in the pores (Fig. 2D3 and D4). These structures were seen to be fully developed, filling the voids after WMTA had been exposed to PBS for several days (Fig. 2D5).

Both iRoot FS and ERRM Putty exposed to PBS exhibited flake-like and acicular structures, as well as spherule clusters, as part of the hydrated cements (Fig. 3A1 - D1 and A2 - D2). Interlinking needle-like crystals formed in the inter-grain spaces, most of which disappeared when iRoot FS and ERRM Putty were exposed to an acidic environment for 14 days. Fragmental laminated structures and amorphous crystal clusters were observed in the voids of the samples after exposure to butyric acid, leaving some empty space. However, the crystallized structures reappeared after exposure to PBS (Fig. 3A5 - D5).

IRM had larger and deeper pores compared to bioceramic materials under the experimental conditions. Moreover, no obvious crystalline structures were observed except on the surface of IRM samples after a 14-day exposure to PBS (Fig. 4B2). These crystal-like structures exhibited main elemental peaks for phosphorus, oxygen, and sodium (Figure not shown). The primary composition of IRM was zinc and oxygen only, indicating that the crystal-like structure could be a PBS precipitate instead of an IRM setting product.

**Fig. 5:**
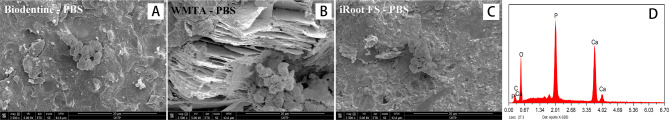
SEM images (**A, B, C,** × 3,500 magnification) of similar spherule cluster on cross-section surface of each bioceramic cement exposed to PBS and EDS analysis indicated almost the same elementary composition **(D)**. Abbreviations: EDS: energy dispersive spectroscope; iRoot FS: iRoot Fast Set Root Repair Material; PBS: phosphate-buffered saline; SEM: scanning electron microscope; WMTA: white mineral trioxide aggregate

According to the EDS analysis, set HCSCs were mainly composed of silica, calcium, oxygen, and a radiopacifier, which showed peaks for bismuth in WMTA and zirconium in Biodentine. The dominant elements of CPSCs were similar to calcium silicate-based cements, with the addition of phosphorus and radiopacifier zirconium, which were detected only in iRoot FS and ERRM Putty. When the test cements were set in PBS or butyric acid, the main elemental composition was almost the same. However, spherule clusters composed mainly of phosphorus, calcium, and oxygen, which indicated the formation of calcium phosphate, were not only displayed in iRoot FS and ERRM Putty samples but also in WMTA and Biodentine specimens exposed to PBS (Fig. 5A - D). EDS analysis displayed the elements contained in crystals with various morphologies (Fig. 2A6, B5, C6, D6 and Fig. 3A6, B6, C6, D6).

### XRD findings

**Fig. 6:**
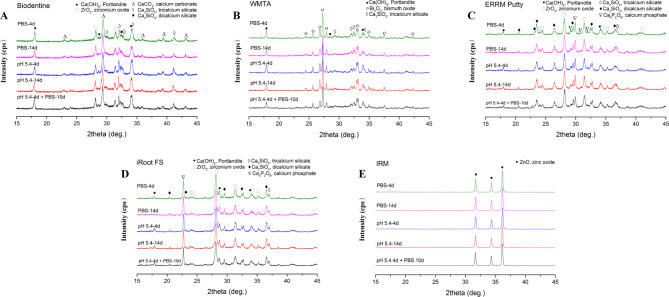
Phase analysis with X-ray diffraction of Biodentine **(A)**, WMTA **(B)**, ERRM Putty **(C)**, iRoot FS **(D)** and IRM **(E)**. Abbreviations: ERRM Putty: EndoSequence Root Repair Material Putty; IRM: intermediate restorative material; iRoot FS: iRoot Fast Set Root Repair Material; WMTA: white mineral trioxide aggregate

The XRD plots in Fig. 6A-E reveal that only the CPSCs (iRoot FS and ERRM Putty) showed detectable peaks of calcium phosphate, whereas the HCSCs (WMTA and Biodentine) did not, even though they were stored in PBS. This may be due to the presence of only trace amounts of the by-product calcium phosphate. The radiopacifier peaks, which correspond to zirconium oxide in ERRM Putty, iRoot FS, and Biodentine and bismuth oxide in WMTA, were well defined compared to other matrix phase peaks. All set bioceramic cements showed the presence of calcium hydroxide (Portlandite) peaks by XRD, which decreased in intensity with longer setting periods. The intensity of some calcium silicate peaks decreased in the materials after longer periods of exposure to PBS, except for iRoot FS, but there was no significant difference in the intensity of these peaks between samples exposed to acid for 4 days and 14 days. Compared to specimens exposed to butyric acid only for 4 days, specimens exposed to PBS for 10 days after the acid showed a decreasing trend in the intensity of these calcium silicate peaks again.

## Discussion

Bioceramic cement applied to the repaired area is often exposed to periodontal tissue that contains pH regulatory systems controlling regional pH [30, 31]. In case of bacterial contamination of this tissue, the local pH might decrease temporarily, but it is expected to return to a slightly alkaline level (pH = 7.4) following endodontic treatment [8]. To replicate this clinical scenario, butyric acid at pH 5.4 was used to create an acidic environment in this research, as it is one of the final products of anaerobic bacterial metabolism that could be taken up by the host [9, 16]. Additionally, PBS at pH 7.4 containing phosphate was chosen to simulate normal tissue fluid conditions in vivo, as in most previous laboratory studies [10, 32]. This was preferred over substitutes such as blood or simulated human plasma fluid, to ensure ethical considerations and control for confounding factors [27, 33]. Samples in subgroup E were stored in contact with acid for four days followed by 10 days of exposure to PBS, to reproduce a clinical scenario where inflammation subsided after four days [34]. It is important to note that when exposed to different environmental conditions, cements undergo a process of hydration and maturation that can cause changes in their physical and chemical properties [32]. In the present study, five cements (WMTA, Biodentine, ERRM Putty, iRoot FS, and IRM) were tested to determine the effects of an acidic environment. Of particular interest is the behavior of iRoot FS in response to acidity, as no prior studies have investigated this aspect. Additionally, the study examines the effects of PBS on changes in cement properties resulting from exposure to low pH.

Microhardness is a crucial parameter for assessing the setting process and overall strength of a material [9]. It measures a material’s resistance to deformation following force application and is closely related to its elastic modulus. Additionally, microhardness is influenced by the stability of a material’s crystal structure and has an inverse correlation with porosity [10, 35, 36]. In this study, dynamic microhardness was evaluated using a Berkovich indenter with a triangular pyramid shape that has an angle of 115 degrees. This was done instead of using the more commonly used Vickers and Knoop indenters, which are two major indenter geometries for hardness testing [37]. The Vickers indenter is shaped like a square pyramid, while the Knoop indenter is an elongated pyramid. As a result, the microhardness values obtained in this study are likely to differ from previously reported data, as other experimental variables were not considered.

The current study assessed the microhardness values of multiple test materials under varying experimental conditions. The study revealed that the microhardness values of the test materials, in descending order, were Biodentine, WMTA, ERRM Putty, iRoot FS, and IRM. Consistent with previous studies [2, 12, 38], IRM exhibited lower microhardness than bioceramic cements, even with different setting conditions and durations. No significant differences were observed between WMTA and ERRM Putty. Biodentine demonstrated superior handling properties compared to WMTA. Both materials required mixing before use, which could lead to the entrapment of air or liquid bubbles. In contrast, ERRM Putty and iRoot FS are hydraulic, pre-mixed, and ready-to-use materials that solidify only in the presence of an aqueous environment. The lower microhardness values of CPSCs, compared to HCSCs, could be attributed to variations in moisture content and consistency beneath the cement surface [12, 39], as well as their distinct compositions and hydration behaviors. However, the details of the reaction mechanism of these CPSCs are not fully clear [2, 40]. Guo et al. [2]. found that the microhardness values of CPSCs (ERRM Putty, iRoot FS) were lower than those of HCSCs (gray and white MTA) when setting in a water bath at 37 °C for 7 and 28 days, but the difference was not significant. Wang et al. [12] also demonstrated, after 7 days of setting in butyric acid at pH 5.4, that the microhardness order from highest to lowest was MTA, ERRM Putty, and IRM (*P* < 0.05), which aligns with our findings.

This study investigated changes in the microhardness and elastic modulus of HCSCs and CPSCs in various environments over time. The findings indicated that all the tested cements showed an increase in microhardness when stored in PBS over time. However, when exposed to an acidic environment, the microhardness values of these cements decreased over time, except for iRoot FS, which exhibited a less pronounced reduction in microhardness. Similar trends were observed for the elastic modulus of the cements. The setting process of iRoot FS appeared to be less affected by butyric acid compared to the other cements, possibly due to differences in their constituents and hydration processes. Although iRoot FS and ERRM Putty are both CPSCs, their hydration processes differ, as confirmed by isothermal calorimeter measurements [2]. Furthermore, iRoot FS has a shorter setting time than ERRM Putty and WMTA, which may make it more resistant to an acidic environment when in constant contact with butyric acid [2]. In contrast, Biodentine is a fast-setting HCSC, and its setting time is shortened by the addition of calcium chloride as an accelerator and polycarboxylate as a water-reducing agent [41]. However, a reduced microhardness of Biodentine was still observed with longer low pH exposure times, unlike iRoot FS. This could be due to differences in the hydration processes between the two cements, although the details of the reaction mechanisms of iRoot FS remain unclear [2]. Previous studies have also reported that acid can have an adverse effect on several physical properties of Biodentine, rather than blood or PBS [32, 42].

Furthermore, the present study showed that the microhardness values were significantly higher when the cements were exposed to PBS (pH 7.4) than when exposed to butyric acid (pH 5.4) at the same setting time. This finding is consistent with previous studies that have reported that acid can lower the microhardness of materials [10–12]. The decrease in microhardness could be explained by the fact that low pH can increase the solubility and porosity of cements, decrease portlandite formation, and potentially inhibit the hydration reaction [16]. However, after 4 days of exposure to acid and 10 days of subsequent exposure to PBS, the microhardness values and elastic modulus of the tested HCSCs and CPSCs increased and even returned to the same level as those of the 4-day PBS exposure. This indicates that storing the cements in PBS can reverse the effect of low pH on the setting process of these cements. Hashem et al. also found that PBS can reverse the bond affected by an acidic environment in MTA [34].

The results of SEM analysis in this study suggested that the acidic environment had an adverse impact on the internal microstructure of both tested HCSCs and CPSCs. Samples stored in acid had more or larger pores than those stored in PBS, consistent with previous studies [9, 12]. Furthermore, with prolonged acid exposure time, the porosity of the cements displayed an increasing trend, although it was not possible to quantitatively compare them or score each characteristic. Lower acidic setting environment also has been confirmed to result in more pores in specimens under SEM observation [12]. The low pH environment groups exhibited fewer or poorly defined crystal clusters, indicating the suppression of crystallization during the hydration reaction because of butyric acid. This inhibition might lead to an unstable cohesive structure and result in lower microhardness [12, 14]. Interestingly, after being kept in contact with PBS following acid exposure, the voids inside the samples were filled with crystal-like structures again, resulting in smaller and less pores. These changes in microstructure could also account for the microhardness variation trend found in this research.

The main components of HCSCs are tricalcium silicate and dicalcium silicate, while in CPSCs, phosphate salts are added based on hydraulic calcium silicates. The hydration behaviors of these substances are expected to vary theoretically, and interpreting the chemical phase transitions in the current testing scenario still poses a challenge. XRD analysis confirmed that calcium phosphate was only detected in CPSCs (iRoot FS and ERRM Putty) rather than HCSCs (WMTA and Biodentine), regardless of the cement setting conditions, even in the samples stored in PBS. This is possibly due to the trace amount of by-product calcium phosphate in hydrated HCSCs, which sets in PBS. Calcium silicate hydrates (CSH) and portlandite (crystalline CH) are the main hydration products of HCSCs [43]. The diffusing calcium and hydroxide ions in HCSCs can react with phosphate salts in PBS and result in a calcium phosphate layer formation [10]. In CPSCs, the same reaction could occur even without exposure to PBS due to the component phosphate salts. However, when butyric acid was used as the setting environment instead of PBS, calcium phosphate formation was theoretically expected only in CPSCs and not in HCSCs. Based on the XRD results presented in Fig. 6, the detection of calcium carbonate in the Biodentine set, bismuth oxide (a radiopacifier) in the WMTA set, and zinc oxide in the IRM set can be attributed to the diverse compositions of these materials, which likely contribute to their unique physicochemical properties. However, the specific mechanisms underlying these observations are still unclear.

In hydrated cements, CSH is not identifiable by XRD due to its poor crystalline structure, while only hydration byproduct CH crystals can be identified [34, 44]. The intensity of each phase in the XRD pattern is proportional to the phase concentration [45]. Within the same setting environment, either butyric acid or PBS, the peaks of portlandite reduced in intensity in all tested bioceramic cements after a longer setting period. It could be speculated that CH was gradually consumed by acid or phosphates over time. Compared to WMTA, CPSCs provide more phosphates, resulting in a lower intensity of portlandite peaks observed in iRoot FS and ERRM Putty. Akhavan et al. reported that after MTA hydration, the intensity of calcium silicate (CS) peaks decreased due to the dissolving of CS reactants and the formation of hydration byproducts [44]. The XRD results in the current study showed that the intensity of CS peaks decreased as exposure to PBS continued over time. However, this intensity did not significantly change between samples exposed to acid for 4 days and 14 days. This information suggested that the hydration process went well in PBS but was hindered by the acidic pH. Nevertheless, once the acidic environment shifted to PBS, a decreasing trend of CS intensity was displayed, suggesting further hydration occurred, in accordance with the results of microhardness and microstructure.

The present in vitro experiment was limited in its ability to fully replicate the clinical local acidic situation, as the realistic inflammatory environment does not maintain a constant level of acidity and the duration of each acidic scenario is uncertain. What’s more, the effect of blood on cements was not considered. Further studies could be performed in the same acidity-basicity experimental settings using extracted human teeth root end filling model to test the material dislocation resistance, sealing ability, bioactivity and so on, in order to provide more laboratory evidence for the selection of root canal repair materials in clinical inflammatory environments.

## Conclusion

Under the conditions of the present study, it can be concluded that an acidic environment adversely affected the physiochemical properties of all materials. When selecting root repair materials, it is important to consider the potential adverse impact they may have in inflammatory conditions. Samples stored in butyric acid exhibited decreasing microhardness and elastic modulus and increasing porosity compared to those exposed to PBS. With prolonged acid exposure, the microhardness and elastic modulus values of HCSCs and CPSCs were reduced, except for iRoot FS, and the bioceramic cements displayed more porous and less crystalline structures. However, these compromises could be reversed by further exposure to PBS. Among the tested cements, Biodentine appears to be the most suitable bioceramic material when supplied to an infected area with an acidic pH.

## Data Availability

The corresponding author can provide the datasets used and/or analyzed during the current study upon reasonable request.
